# Developmental Regulation of KCC2 Phosphorylation Has Long-Term Impacts on Cognitive Function

**DOI:** 10.3389/fnmol.2019.00173

**Published:** 2019-07-23

**Authors:** Yvonne E. Moore, Leslie C. Conway, Heike J. Wobst, Nicholas J. Brandon, Tarek Z. Deeb, Stephen J. Moss

**Affiliations:** ^1^Department of Neuroscience, Tufts University School of Medicine, Boston, MA, United States; ^2^AstraZeneca-Tufts University Laboratory for Basic and Translational Neuroscience Research, Tufts University School of Medicine, Boston, MA, United States; ^3^Neuroscience, R&D Biopharmaceuticals, AstraZeneca, Boston, MA, United States; ^4^Department of Neuroscience, Physiology and Pharmacology, University College London, London, United Kingdom

**Keywords:** KCC2, depolarizing GABA, autism, cognition, social behavior, memory, phosphorylation

## Abstract

GABA_A_ receptor-mediated currents shift from excitatory to inhibitory during postnatal brain development in rodents. A postnatal increase in KCC2 protein expression is considered to be the sole mechanism controlling the developmental onset of hyperpolarizing synaptic transmission, but here we identify a key role for KCC2 phosphorylation in the developmental E_GABA_ shift. Preventing phosphorylation of KCC2 *in vivo* at either residue serine 940 (S940), or at residues threonine 906 and threonine 1007 (T906/T1007), delayed or accelerated the postnatal onset of KCC2 function, respectively. Several models of neurodevelopmental disorders including Rett syndrome, Fragile × and Down’s syndrome exhibit delayed postnatal onset of hyperpolarizing GABAergic inhibition, but whether the timing of the onset of hyperpolarizing synaptic inhibition during development plays a role in establishing adulthood cognitive function is unknown; we have used the distinct KCC2-S940A and KCC2-T906A/T1007A knock-in mouse models to address this issue. Altering KCC2 function resulted in long-term abnormalities in social behavior and memory retention. Tight regulation of KCC2 phosphorylation is therefore required for the typical timing of the developmental onset of hyperpolarizing synaptic inhibition, and it plays a fundamental role in the regulation of adulthood cognitive function.

## Introduction

Cl^−^-permeable Glycine and GABA_A_ receptors are the exclusive mediators of fast synaptic inhibition in the central nervous system. Maintaining low intraneuronal Cl^−^ levels, and thus an inwardly directed electrochemical driving force for Cl^−^, is essential for the inhibitory efficacy of these receptors (Bormann et al., 1987). However, during the early postnatal period, neurons have a high [Cl^−^]_i_, resulting in depolarizing responses to GABA_A_ receptor activation, demonstrated through studies on the rodent brain (Ben-Ari et al., 1989, 2007; Owens et al., 1996). A progressive increase in Cl^−^ extrusion as development proceeds is responsible for the conversion of GABA_A_ currents from depolarizing to hyperpolarizing (Rivera et al., 1999). This increase in Cl^−^ extrusion is widely considered to be due to the functional upregulation of the neuron-specific K^+^/Cl^−^ cotransporter type 2 (KCC2), which couples the outwardly directed K^+^ gradient to extrude Cl^−^ from the cell against its concentration gradient (Li et al., 2002; Uvarov et al., 2006). KCC2 activity is therefore essential for establishing the developmental onset of hyperpolarizing GABA_A_ receptor currents.

An upregulation of KCC2 gene expression and protein production are currently thought to be the primary driver of the increase in KCC2 function during development. However, we have previously demonstrated a critical role for the phosphorylation of several KCC2 residues within its intracellular C-terminal domain that regulate its function in the mature brain. Phosphorylation of residue serine 940 (KCC2-S940) is necessary for maintaining KCC2 function under pathological conditions in the adult brain (Lee et al., 2007, 2011; Silayeva et al., 2015). In contrast, phosphorylation of threonine residues 906 and 1007 (KCC2-T906/T1007) decreases during development (Rinehart et al., 2009; Friedel et al., 2015), and simultaneous alanine substitution at both residues increases KCC2 function in cell lines and mature neurons (Rinehart et al., 2009; de Los Heros et al., 2014; Titz et al., 2015; Moore et al., 2018). But it is unknown whether phosphorylation of these residues similarly regulates KCC2 function during development, and subsequently the timing of the developmental onset of hyperpolarizing fast synaptic inhibition.

Importantly, KCC2 dysfunction and dysregulation of Cl^−^ homeostasis occurs in neurodevelopmental disorders including Down syndrome (Deidda et al., 2015), fragile × syndrome (He et al., 2014), and Rett syndrome (Duarte et al., 2013; Tang et al., 2016). Postnatal E_GABA_ hyperpolarization is delayed in these disorders, but whether this contributes to any of the cognitive deficits characteristic of these pleiotropic disorders is unknown. We, therefore, sought to identify whether the amount of KCC2 function and the timing of the developmental onset of fast synaptic inhibition plays a determining role in cognitive function in adulthood, particularly in the context of behaviors associated with autism-spectrum disorders.

## Materials and Methods

### Animal Care

All animal studies were performed with protocols approved by the Institutional Animal Care and Use committee of Tufts New England Medical Center. Animals live in temperature-controlled rooms on a 12 h day/light cycle, fed *ad libitum*, with cage changes performed twice weekly.

### Tissue Preparations

#### Cultured Mouse Hippocampal Neurons

Electrophysiology experiments were performed on hippocampal neurons cultured from P1 mouse pups. Pups were cooled on ice before decapitation and brain removal. Brains were submerged in ice-cold HEPES-buffered saline solution, meninges removed from the brain surface, and hippocampi dissected out. Hippocampi were transferred to 10 mL of 0.25% trypsin in HBSS at 37°C for 9 min to dissociate the tissue. Trypsin was then removed from the hippocampi, which were then washed three times with HBSS to remove any residual trypsin. Ten milliliters of fresh media was then added to the cells, and the cells were triturated with a 10 mL pipette, by gently pipetting up and down 15 times. Cells were then filtered to remove non-dissociated tissue. Four-hundred thousand cells were plated onto each pre-prepared PLL coated 30 mm dish each containing 3 mL culture media [Neurobasal A media containing B27 (2%), glucose (0.6%), Glutamax (1%), and penicillin/streptomycin (1%)]. Cells were maintained at 37°C in a humidified 5% CO_2_ incubator.

#### Cultured Rat Hippocampal Neurons

Cultured hippocampal neurons obtained from Sprague-Dawley rat embryonic day 18 embryos. Neuronal preparation was carried out as described above for the mouse neurons. Cells were then plated onto pre-prepared PLL coated 6cl dishes, each containing 6 mL of culture media [Neurobasal media containing B27 (2%), glucose (0.6%), Glutamax (1%), and penicillin/streptomycin (1%)]. Cells were maintained at 37°C in a humidified 5% CO_2_ incubator. Hippocampal neurons were lysed in RIPA Buffer (2% Triton-X-100, 0.5% deoxycholic acid, 5 mM EDTA, 5 mM EGTA, 1 mM sodium orthovanadate, 25 mM sodium fluoride, 10 mM sodium pyrophosphate, 100 mM NaCl, 10 mM sodium phosphate monobasic, pH7.4) containing protease inhibitors by rotating samples for 30 min at 4°C. Insoluble material was removed by centrifugation for 15 min at 15,700× *g*.

#### Whole Hippocampal Dissection

Mice were deeply anesthetized with isoflurane before decapitation at the second cervical vertebrae. Brains were carefully removed and rinsed in ice-cold phosphate buffered saline. The hippocampus were then dissected with forceps, and immediately put into ice-cold RIPA lysis buffer (2% Triton-X-100, 0.5% deoxycholic acid, 5 mM EDTA, 5 mM EGTA, 1 mM sodium orthovanadate, 25 mM sodium fluoride, 10 mM sodium pyrophosphate, 100 mM NaCl, 10 mM sodium phosphate monobasic, pH7.4) containing protease inhibitors. Here, the tissue was dissociated with a 26G needle and rotated a 4°C for 30 min to lyse. The samples were then centrifuged at 15,700× *g* for 15 min at 4°C to pellet all insoluble material. The supernatant was used for western blotting.

### Biochemistry

#### SDS-PAGE and Western Blotting

Lysate samples were separated by SDS-PAGE and transferred to nitrocellulose membrane. Following the transfer, membranes were blocked at room temperature for 1 h in 5% milk, 1% BSA in PBS-Tween. Membranes were then incubated with primary antibody diluted in blocking solution overnight at 4°C to detect total KCC2 (Millipore 07–432), KCC2 pS940 [PhosphoSolutions, previously characterized (Lee et al., 2011)], and α-tubulin (Abcam 7291). Membranes were then washed three times in PBS-Tween and incubated with the respective HRP-conjugated secondary antibody. Secondary antibody incubations were carried out in blocking solution for 1 h at room temperature. Membranes were then washed three times in PBS-Tween, followed by one wash in PBS. Chemiluminescence signal was detected using SuperSignal West Dura Extended Duration Substrate (Thermo Scientific). Quantification of chemiluminescence signal was carried out using Image Lab 5.0 (BioRad).

### Patch Clamp Electrophysiology

All electrophysiology experiments were performed on hippocampal mouse neurons cultured from P1 pups. All data incorporates Ns from a minimum of three separate neuronal dissections per genotype.

#### Solutions and Set Up

Recordings were conducted at 32° C in bath saline containing (in mM) NaCl 140, KCl 2.5, CaCl_2_ 2, MgCl_2_ 1.5, Hepes 10, glucose 11, pH 7.4 NaOH. TTX was used at a concentration of 500 nM. Muscimol was used at 1μM and applied through a three-barrel microperfusion system (700 μm, Warner Instruments, Hamden, CT, USA) closely positioned above the cell, at a rate of 0.5 mL/min and we used a computer-controlled perfusion fast-step device (Warner Instruments, Hamden, CT, USA) to ensure fast and complete exchange of solutions. Perforated patch clamp experiments were performed with gramicidin (50 μg/mL) inside a patch pipette (3–6 mohm) containing (in mM) KCl 140, HEPES 10, pH 7.4 KOH, and experiments began once adequate perforation had been achieved, classed as a series resistance <50 mohm.

#### E_GABA_ Measurements

E_GABA_ was measured by application of muscimol (1 μM) during positive going voltage ramps (10 mV or 20 mV, 1 s duration), and subsequent calculation of the reversal potential of the leak-subtracted muscimol currents. Data were acquired at 10 kHz with an Axopatch 200B amplifier and Clampex 10 software (Molecular Devices, Sunnyvale, CA). Once E_GABA_ values were obtained, these were used to determine the intracellular Cl^−^ concentration of the cells. This was done using the Nernst equation: ${\text{E}}_{\text{Cl}-}=\text{RT/zF}\times \ln{\left[Cl^{-}\right]}_{\text{o}}/{\left[Cl^{-}\right]}_{\text{i}}$.

### Behavior

For all behavioral experiments, mice were habituated in the testing rooms for 1 h prior to testing. Testing was performed at similar times of the day (between 9 and 11 am), in temperature-controlled rooms (70–74°C). Littermates were always tested at the same time. Following completion of each experiment, mice were returned to their home cage. Equipment was cleaned between each mouse using 70% ethanol, followed by Clidox (chlorine dioxide based sterilant). Male mice were used for all experiments.

#### 3-Chamber Social Interaction Assay

Mice were tested in this assay between 6 and 7 weeks of age. A 3-chamber set up, each chamber measuring 40 cm × 40 cm, was used. Mice were placed in the center chamber, and allowed to explore all chambers for 10 min. Metal cages, measuring 4 cm × 4 cm × 5 cm with 1 cm gaps between each vertical bar, were then placed in the center of the left and right chambers, and a juvenile unfamiliar male mouse (3–4 weeks old) placed under one of the cages. The test mice were then allowed to explore the arena for 10 min and time spent in the chamber with the mouse vs. the empty cage was calculated. An additional unfamiliar juvenile male mouse was then placed under the previously empty cage, and the test mice were again allowed to explore the arena for 10 min and the time spent in the chamber with the familiar vs. the novel mouse was calculated. An overhead camera and Ethovision software was used to detect time spent in each region of the arena, and to generate heat maps of the data.

#### Barnes Maze Assay

Mice were tested in this assay between 8 and 10 weeks of age. The maze was a circular platform, measuring 1.5 m in diameter, with 40 holes at the perimeter each measuring 2.5 cm in diameter. An escape tunnel was placed under one hole. Different shapes drawn onto article were used as spatial cues around the maze. Mice were placed onto the center of the maze under a wire cage and allowed to orient themselves for 10 s. The cage was then lifted and the mice were given 3 min to explore the maze and find the escape hole, and latency to exit the maze through this escape tunnel was recorded. If the mice did not enter the escape within 3 min they were removed from the maze and their time was recorded as the maximum 180 s. This was repeated twice on each day, with a 30 min delay between each trial, and the average of these trials was used as the final value. This process was completed on six consecutive days. To assess memory, on day 7 the escape hole was removed, and the mice were given 3 min to explore the arena. The time spent at each hole was measured, and each hole assigned to a 45° bin each composed of five holes, with 0° relating to the goal hole and the two holes either side of this hole. The 45° bins then continued in a clockwise direction around the perimeter of the maze. This was also repeated on day 14 to memory after 1 week of no exposure to the maze. An overhead camera and Ethovision software was used to detect time spent in each region of the arena, and to generate heat maps of the data.

### Statistical Analysis

All data are presented as the mean ± standard error of the mean (SEM). Biochemistry data were analyzed using the one-way analysis of variance (ANOVA) test. Electrophysiology data were analyzed using the unpaired *T*-test. Behavioral data were analyzed using the unpaired *T*-test to compare between genotypes, and the paired *T*-test to compare data obtained on individual mice across different trials. *P* values < 0.05 are considered statistically significant.

## Results

### Phosphorylation of KCC2 Residues S940 and T906/T1007 Control the Timing of the Postnatal Onset of Hyperpolarizing Inhibition

**Figure 1:**
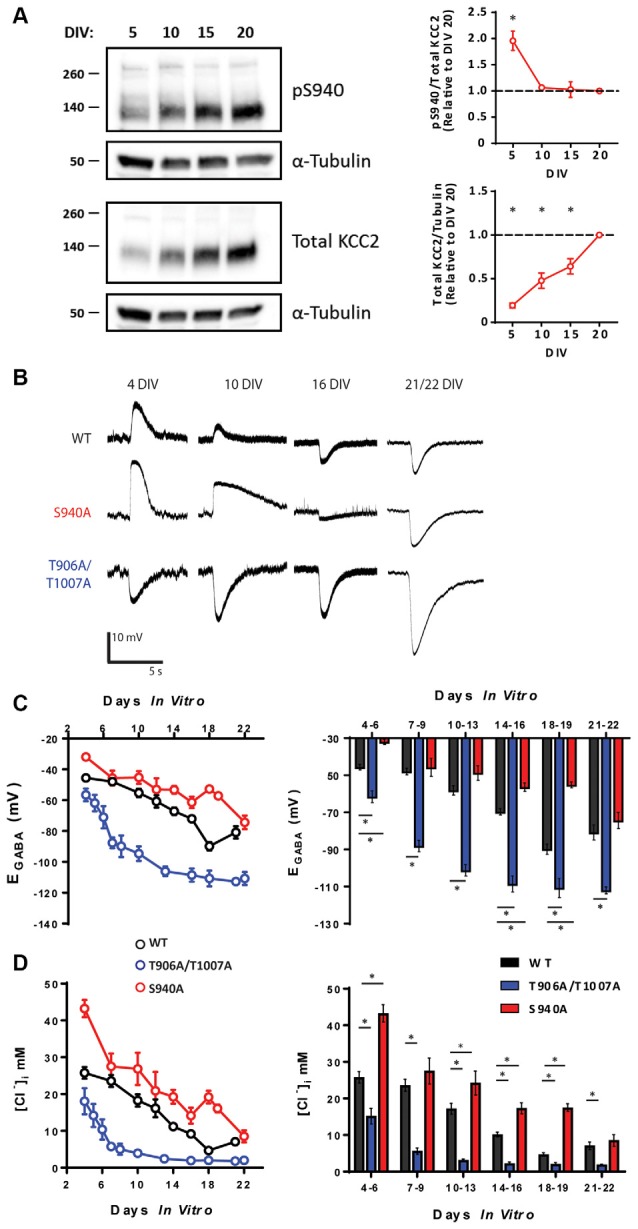
KCC2-S940 and T906/T1007 phosphorylation orchestrate the postnatal upregulation of KCC2 function. **(A)** Western blotting was used to assess developmental changes in KCC2 expression and KCC2-S940 phosphorylation in cultured hippocampal neurons at 5, 10, 15 and 20 DIV. Levels of KCC2 expression and S940 phosphorylation are expressed relative to 20 DIV. KCC2 expression progressively increased between 5 and 20 DIV. Relative to levels of KCC2 expression, S940 phosphorylation decreased between 5 and 10 DIV but was followed by maintenance at this level as the neurons further matured. **(B)** The polarity of GABA_A_ currents was recorded from WT, S940A and T906A/T1007A cultured mouse neurons between 4 and 22 DIV by activating GABA_A_ receptors using brief muscimol application. WT and S940A neurons displayed depolarizing GABA_A_ currents at the earliest stages of neuronal development but shifted to hyperpolarizing responses by 16 DIV. In contrast, T906A/T1007A neurons displayed hyperpolarizing GABA_A_ currents as early as 4 DIV. **(C)** E_GABA_ measurements were obtained from WT, S940A and T906A/T1007A neurons between 4 and 22 DIV. A progressive negative E_GABA_ shift was detected in WT neurons as development proceeded. In contrast, this negative E_GABA_ shift was delayed in the S940A neurons and accelerated in the T906A/T1007A neurons. E_GABA_ values were also binned into groups of 2–4 DIV to enable statistical analysis. The corresponding [Cl^−^]_i_ measurements were also calculated **(D)**. *Statistically significant (*p* < 0.05) for the indicated comparisons, see text and Supplementary Tables 1 and 2 for exact *p* values.

Phosphorylation of KCC2 residue serine 940 (S940) regulates KCC2 function in the adult brain (Lee et al., 2007; Silayeva et al., 2015), and we sought to examine the developmental profile of S940 phosphorylation. We measured total KCC2 expression and KCC2 S940 phosphorylation in cultured rat hippocampal neurons at 5, 10, 15 and 20 days *in vitro* (DIV) and detected phosphorylation of this residue at each of these time points. Total KCC2 expression increased linearly over-development, with significantly lower expression at 5 DIV (Mean: 0.19 a.u ± 0.02., compared to 20 DIV, *P* < 0.0001, *N* = 3), 10 DIV (Mean: 0.48 a.u ± 0.05., compared to 20 DIV, *P* < 0.0001, *N* = 3) and 15 DIV (Mean: 0.64 a.u ± 0.05., compared to 20 DIV, *P* = 0.0003, *N* = 3). We detected a decrease in S940 phosphorylation between 5 and 10 DIV, followed by maintenance at this level as the neurons further matured (Figure 1A; 5 DIV Mean: 1.96 a.u. ± 0.18, *P* = 0.0012 compared to 20 DIV, *N* = 3; 10 DIV Mean: 1.06 a.u. ± 0.03, *P* = 0.96 compared to 20 DIV, *N* = 3; 15 DIV Mean: 1.03 a.u. ± 0.15, *P* = 1.00 compared to 20 DIV *N* = 3). Given that S940 phosphorylation scales with the developmental increase in total KCC2 expression between 10 and 20 DIV, we hypothesized that S940 phosphorylation plays a role in the developmental regulation of KCC2 function. To answer this question, we performed gramicidin perforated patch-clamp experiments to measure E_GABA_ values between 4 and 22 DIV on hippocampal neurons cultured from phospho-mutant knock-in mice, in which residue S940 is mutated to an alanine residue (S940A) to prevent its phosphorylation (Silayeva et al., 2015; Figure 1B). Compared to WT, the developmental onset of hyperpolarizing GABAergic inhibition was delayed in the S940A neurons, deviating from WT values between 4 and 6 DIV and 14 and 18 DIV (Supplementary Table S1), but displayed similar E_GABA_ values to WT neurons by 21–22 DIV (Figure 1C; Supplementary Table S2). [Cl^−^]_i_ values were calculated using the Nernst equation (Figure 1D; Supplementary Table S2), and S940A neurons had higher [Cl^−^]_i_ levels between 4 and 6 DIV and 10 and 18 DIV. This indicates that S940 phosphorylation plays an important role in regulating KCC2 function at several stages of postnatal development and highlights a critical role for KCC2-S940 phosphoregulation for the appropriate timing of the developmental onset of hyperpolarizing synaptic inhibition. Resting membrane potentials were also measured across development and no differences were detected between WT and S940A neurons (Supplementary Figure S1; Supplementary Table S3).

KCC2 can also be phosphorylated at threonine residues 906 and 1007 (T906/T1007; Rinehart et al., 2009). Phosphorylation of these residues decreases during development which may contribute to the developmental upregulation of KCC2 function (Rinehart et al., 2009; Friedel et al., 2015). Using an additional phospho-mutant knock-in mouse model, in which KCC2-T906/T1007 residues are mutated to alanine to prevent their phosphorylation (T906A/T1007A; Moore et al., 2018), we were able to directly assess the role of these phosphorylation sites in the regulation of KCC2 function during development. We performed gramicidin perforated patch-clamp experiments on hippocampal neurons cultured from T906A/T1007A mice and measured E_GABA_ between 4 and 22 DIV (Figure 1B). E_GABA_ values were strongly hyperpolarized in T906A/T1007A neurons compared to WT neurons across all stages of neuronal development (Supplementary Table S1). The corresponding [Cl^−^]_i_ values were also measured, demonstrating [Cl^−^]_i_ levels were lower in the T906A/T1007A neurons at all time points (Figure 1C; Supplementary Table S2). This indicates that preventing phosphorylation of these sites significantly accelerates the postnatal onset of hyperpolarizing inhibition, largely eliminating postnatal depolarizing GABAergic signaling. Resting membrane potentials were also measured across development and no differences were detected between WT and T906A/T1007A neurons (Supplementary Figure S1; Supplementary Table S3).

KCC2-T906A/T1007A and KCC2-S940A mutations altered KCC2 function independently of KCC2 protein expression in mature neurons. To determine if this is also the case in immature neurons, we compared KCC2 expression in total hippocampal lysate of P5 WT (Mean: 2.99 ± 0.27 a.u.; *N* = 3), T906A/T1007A (Mean: 3.69 ± 0.35 a.u.; *N* = 3) and S940A (Mean: 2.82 ± 0.37 a.u.; *N* = 3) mice (Supplementary Figure S2). No difference in KCC2 expression levels was detected between WT and T906A/T1007A (*P* = 0.19) or between WT and S940A (*P* = 0.73) suggesting that these mutations are specifically enhancing the surface activity of KCC2 rather than by altering KCC2 expression during this time period.

### KCC2 Function Alters Social Interaction Behaviors

**Figure 2:**
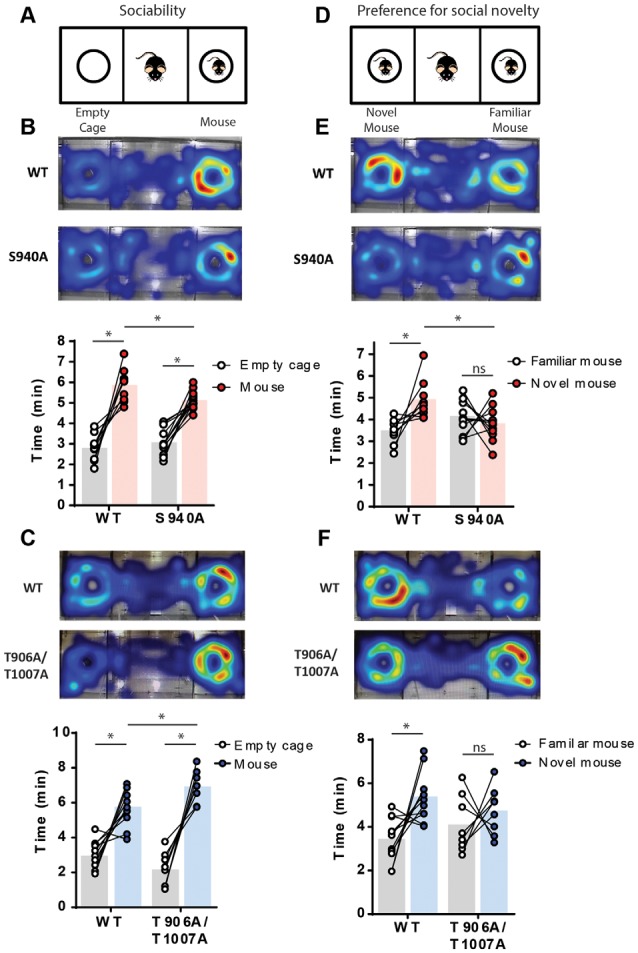
Both delayed and accelerated developmental onset of hyperpolarizing inhibition impact social behavior. A 3-chamber social interaction assay was used to assess sociability and preference for social novelty in the S940A and T906A/T1007A mice. Heat maps indicate the predominate regions of exploration within the 3 chambers (warm colors indicate longer time spent than cooler colors). **(A)** Diagram of the sociability assay. Mice were allowed to explore either an unfamiliar mouse or an empty cage. **(B)** Both S940A mice and their WT littermates spent more time interacting with the mouse vs. the empty cage, but the S940A mice spent less time interacting with the mouse than their WT littermates did, suggesting a mild sociability deficit in the S940A mice. **(C)** T906A/T1007A mice and their WT littermates both showed preference for interacting with the mouse vs. the empty cage, but the T906A/T1007A mice spent more time with the mouse than the WT mice, suggesting that the T906A/T1007A mice are more social. **(D)** Diagram of the preference for social novelty assay. Immediately after the sociability test, an additional unfamiliar mouse was placed under the empty cage and mice were allowed to explore both of these mice. **(E)** S940A mice showed no preference for interacting with the novel mouse, unlike their WT littermates. **(F)** T906A/T1007A mice showed no preference for interacting with the novel mouse, unlike their WT littermates. *Statistically significant (*p* < 0.05) for the indicated comparisons, see text for exact *p* values. ns, no significant change.

Delayed timing of the developmental onset of fast synaptic inhibition has been detected in several models of neurodevelopmental disorders associated with autism-like behaviors (Duarte et al., 2013; He et al., 2014; Deidda et al., 2015; Tang et al., 2016). Social interaction deficits that persist into adulthood are a core feature of autism-spectrum disorders, and so we assessed the impact of the S940A or the T906A/T1007A mutations on social behavior in adult mice (6–7 weeks old). Social interaction was assessed using a 3-chamber social interaction test. Firstly, sociability was examined by measuring time spent interacting with an unfamiliar mouse vs. an empty cage (Figure 2A). S940A mice, and their WT littermates, both spent more time interacting with the mouse vs. the empty cage (Figure 2B; WT: Empty 2.81 ± 0.20 min, Mouse 5.88 ± 0.25 min, *p* < 0.0001, *N* = 10; S940A: Empty 3.1 ± 0.2 min, Mouse 5.1 ± 0.1 min, *p* < 0.0001, *N* = 13). However, the S940A mice spent less total time interacting with the mouse compared to their WT littermates (WT: 5.9 ± 0.3 min; S940A: 5.1 ± 0.1 min; *p* = 0.0075), indicating that S940A mice have a reduced preference for social interaction. Although, time spent near the empty cage was not significantly different between WT and S940A mice (WT: 2.81 ± 0.2 min; S940A: 3.08 ± 0.2 min; *p* = 0.3135). T906A/T1007A mice also spent more time interacting with the mouse vs. the empty cage (Figure 2B; Empty: 2.2 ± 0.4 min, Mouse: 7.0 ± 0.3 min; *p* = 0.0002, *N* = 8), as did their WT littermates (Empty: 3.0 ± 0.3 min, Mouse: 5.8 ± 0.3 min; *p* = 0.0005, *N* = 11). Interestingly, the T906A/T1007A showed increased sociability compared to WT mice, demonstrated by an increase in the total time spent with the mouse compared to WT (Figure 2C; WT: 5.8 ± 0.3 min; T906A/T1007A: 7.0 ± 0.3 min; *p* = 0.0247). Moreover, T906A/T1007A mice trended toward spending less time near the empty cage than WT mice, but not to a significant degree (WT: 2.98 ± 0.2 min; T906A/T1007A: 2.18 ± 0.3 min; *p* = 0.0688).

A novel mouse was then placed in the empty cage to assess preference for social novelty (Figure 2D). S940A mice showed no preference for interacting with the novel mouse compared to the familiar mouse (Familiar: 4.13 ± 0.02 min, Novel: 3.8 ± 0.2 min; *P* = 0.42, *N* = 12), in contrast to their WT littermates which did show preference for the novel mouse (Figure 2E; Familiar: 3.5 ± 0.6 min, Novel: 4.9 ± 0.3 min; *P* = 0.0160, *N* = 9). WT mice also spent greater total time interacting with the novel mouse compared to S940A mice (WT: 4.9 ± 0.3 min; S940A: 3.8 ± 0.2 min; *P* < 0.0063). Similarly, the T906A/T1007A mice showed no preference for the novel mouse (Familiar: 4.1 ± 0.5 min, Novel: 4.7 ± 0.4 min; *p* = 0.46, *N* = 8), while their WT littermates did show preference for the novel mouse (Figure 2F; Familiar: 3.4 ± 0.3 min, Novel: 5.38 ± 0.01 min; *P* = 0.0112, *N* = 11). These data demonstrate that either delaying or accelerating the developmental E_GABA_ shift results in altered social behaviors, indicating that the postnatal onset of KCC2 function must be finely orchestrated to establish typical social behaviors in adulthood.

### KCC2 Function Impacts Spatial Memory

**Figure 3:**
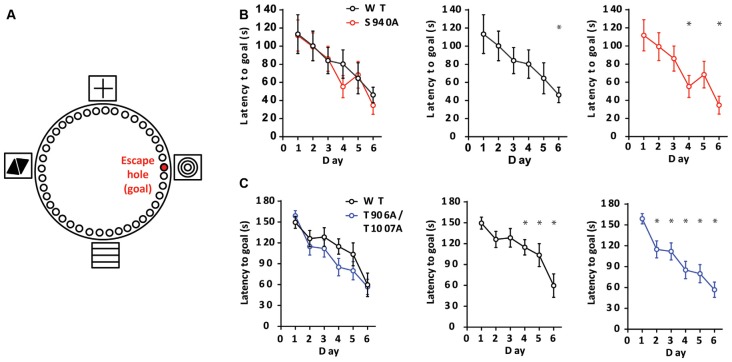
KCC2 phosphorylation can impact rate of spatial learning. **(A)** Diagram of the Barnes maze assay used to assess spatial learning in the mutant mice. Mice were exposed to the maze over six consecutive days, learning to associate the location of an escape hole with a spatial cue (concentric circles). Additional spatial cues were placed around the maze as depicted. **(B)** Latency to enter the goal hole was measured on days 1–6, and the S940A mice showed no significant differences in time to goal hole compared to WT littermates on any of these days. Rate of learning (day in which there is a significant reduction in latency to goal compared to that of day 1) was also comparable. **(C)** Latency to enter the goal hole was measured on days 1–6, and the T906A/T1007A mice showed no significant differences in time to goal hole compared to WT littermates on any of these days. However, rate of learning (day in which there is a significant reduction in latency to goal compared to that of day 1) was accelerated in the T906A/T1007A mice. *Statistically significant (*p* < 0.05) for the indicated comparisons, see Supplementary Tables 4 and 5 for exact *p* values.

We next sought to determine if altered timing of developmental E_GABA_ hyperpolarization impacts learning and memory, as intellectual disabilities are frequently present in patients with autism-spectrum disorders. To assess learning and memory we used a Barnes maze assay, and latency to enter the escape hole over six consecutive days was recorded (Figure 3A). The S940A mice showed comparable escape latencies to their WT littermates over the 6-day learning period (WT: *N* = 11; S940A: *N* = 12 for all days; Figure 3B; Supplementary Table S4). Similarly, the T906A/T1007A mice did not exhibit differences in the latency to find the goal hole compared to WT littermates (WT: *N* = 14; T906A/T1007A: *N* = 15 for all days; Figure 3C; Supplementary Table S5). However, the T906A/T1007A mice did show a significant improvement over their day 1 escape latencies by training day 2. In comparison, their WT littermates which did not show significantly improved performance over their day 1 escape latencies until training day 4. This suggests that rate of spatial learning was mildly improved in the T906A/T1007A mice (Figure 3C; Supplementary Table S5).

**Figure 4:**
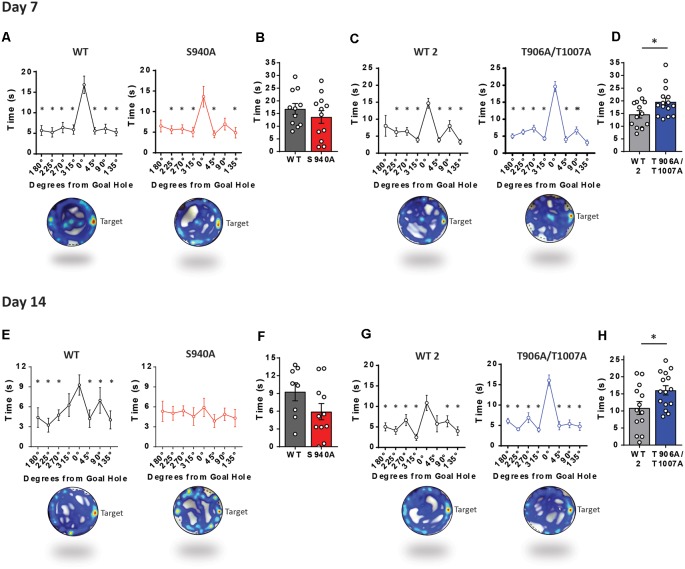
KCC2-S940A and KCC2-T906A/T1007A mutations have bidirectional effects on spatial memory retention. Spatial memory was assessed using a Barnes maze assay. After 6 days of learning the location of an escape hole (see Figure 3) time spent at each of the holes was measured on day 7 and day 14 upon removal of the escape tunnel, and data binned into 45° groups. **(A)** S940A mice and their WT littermates both show preference for the goal area on day 7, and spent comparable time at the goal area as their WT littermates **(B)**. **(C)** T906A/T1007A mice and their WT littermates both show preference for the goal area on day 7, but T906A/T1007A mice spent more time at the goal than their WT littermates **(D)**. **(E)** S940A mice show no preference for the goal hole on day 14, but time spent at the goal was comparable to their WT littermates **(F)**. **(G)** T906A/T1007A mice show enhanced specificity of the spatial memory on day 14 compared to their WT littermates, and T906A/T1007A mice spent more time at the goal than their WT littermates **(H)**, indicating that the T906A/T1007A mutations enhance spatial memory retention. *Statistically significant (*p* < 0.05) for the indicated comparisons, see Supplementary Table 6 for exact *p* values. ns, no significant change.

Long term memory was then assessed over increasing periods of time after the learning portion of the Barnes maze assay. We removed the escape chamber on day 7 and 14 and then measured the time spent in the space where the escape hole was originally located. Time spent at the goal hole was comparable between WT and S940A mice on both day 7 (WT: *N* = 11; S940A: *N* = 12; Figures 4A,B) and day 14 (WT: *N* = 8; S940A: *N* = 11; Figures 4E,F) which would initially suggest no memory impairment is present in S940A mice. However, when memory retention is normal, mice will spend significantly more time investigating the goal hole than any of the remaining holes in the maze. In other words, they will display a specific interest in the goal hole over all other holes. Interestingly, by day 14 S940A mice spent equal amounts of time at the non-goal regions as they did at the goal region demonstrating a lack of spatial memory specificity compared to their WT littermates, suggesting that S940A mice have a deficit in memory retention (Figure 4E; Supplementary Table S6). In contrast, T906A/T1007A mice spent more time at the goal hole position at both day 7 (WT: *N* = 14; T906A/T1007A: *N* = 15; Figures 4C,D) and day 14 (WT: *N* = 13; T906A/T1007A: *N* = 15; Figures 4E,F) compared to their WT littermates (Supplementary Table S6), suggesting that increasing KCC2 function may improve memory retention.

## Discussion

KCC2 is heavily phospho-regulated in the adult brain (Silayeva et al., 2015; Moore et al., 2017, 2018) and we have now identified a critical role for phosphorylation in regulating KCC2 function during postnatal brain development. We have determined that KCC2 phospho-regulation at sites S940 and T906/T1007 plays differential roles in cognition. Our data suggest a prolonged postnatal period of depolarizing GABA that may contribute to behavioral manifestations of neurodevelopmental disorders, specifically abnormalities in social behavior and memory retention. Unexpectedly, accelerating the postnatal onset of KCC2 function also resulted in social abnormalities, indicating that postnatal depolarizing GABA may be necessary for the development of typical social behavior. However, KCC2 gain-of-function mice excelled at spatial memory tasks suggesting that increasing KCC2 activity can also positively impact cognitive function.

The time-course of the developmental E_GABA_ shift we observed in WT hippocampal neurons matches the timing of the disappearance of giant depolarizing potentials in CA3 slices (Ben-Ari et al., 1989), the E_GABA_ shift in CA1 neurons in slices (Zhang et al., 1991) and cortical neurons of the rat (Owens et al., 1996), all of which indicate that the mechanisms underlying the E_GABA_ shift is preserved in culture. By preventing the phosphorylation of endogenous KCC2 *in vivo* at sites S940 and T906/T1007 we were able to precisely establish the postnatal periods during which phosphorylation of these residues plays a role in the developmental onset of hyperpolarizing synaptic inhibition. KCC2 was most heavily phosphorylated at site S940 during the 1st postnatal week, and the largest deficit in KCC2 function in the S940A neurons was detected at this time period. This indicates that S940 phosphorylation plays an important role in shaping the timing of the postnatal onset of hyperpolarizing inhibition. The S940A mutation had less impact on basal KCC2 function as the neurons matured and had no detrimental impact by the most mature stages of development. Interestingly, the impact of the S940A mutation on KCC2 function followed a biphasic pattern, with no effect on basal KCC2 function detected between WT and S940A neurons at both 7–9 DIV and 21–22 DIV, despite deficits detected at all other developmental time points. Interpreting this finding is complicated by our lack of understanding of the signaling cascades that regulate S940 phosphorylation during development, and whether or not these pathways also change during development. S940 is phosphorylated by protein kinase C in mature neurons (Lee et al., 2007), which may provide a starting point for future investigations into the signaling cascades that impinge on KCC2-S940 and affect its function. G-protein-coupled receptor (GPCR) signaling through group 1 metabotropic glutamate receptors, metabotropic Zn(2+) receptors, 5-HT2A serotonin receptors and A3A-type adenosine receptors can regulate KCC2 function in neurons (Mahadevan and Woodin, 2016), and so examining these signaling cascades in the context of developmental KCC2 regulation may be informative. In contrast, phosphorylation of KCC2 at sites T906/T1007 appears to play a key role in regulating KCC2 function at all stages of neuronal development. Interestingly, elimination of depolarizing GABA occurred as early as 4 DIV in the T906A/T1007A neurons. KCC2 protein expression is still very low at such early stages of development which strongly suggests that the phosphorylation state of KCC2 is the major determinant of the efficacy of KCC2-mediated Cl^−^ transport, surpassing the importance of KCC2 protein expression levels.

Recent work has identified that GABA is depolarizing for an extended period of time in several neurodevelopmental disorders associated with autism-like behaviors (He et al., 2014; Deidda et al., 2015; Tang et al., 2016). Whether this delayed E_GABA_ shift plays any role in the behavioral consequences of these pleiotropic disorders has not previously been assessed, but using the S940A mice we were able to address this question. Abnormal social behavior is a core feature of autism-spectrum disorders, and we detected profound social interaction abnormalities in the S940A mice. This interesting finding suggests that the delayed onset of hyperpolarizing synaptic inhibition detected in various models of neurodevelopment disorders associated with social interaction deficits may, in fact, be responsible for this abnormal behavior. Correcting this delay in established models of autism-spectrum disorders would determine whether this delay is solely responsible for the abnormal social behavior.

It was particularly interesting that premature onset of KCC2 function also results in abnormal social behavior, but increased preference in social interaction in contrast to a reduced preference seen in the S940A mice. This suggests that reduced vs. increased KCC2 activity alters the function of neuronal networks involved in social behaviors in different ways. However, T906A/T1007A mice did display deficient preference for social novelty, which is a characteristic behavior seen in mouse models of autism-spectrum disorders (Takumi et al., 2019). This was an unexpected finding as no previous studies have linked excessive KCC2 function to the onset of autism-like behaviors. This is especially important as enhancing KCC2 function has been proposed as a novel mechanism for treating seizures (Moore et al., 2018), but our finding here raises some questions regarding the potential safety of using pharmacological activators of KCC2 in healthy individuals during early brain development.

In addition to the impact of the S940A and T906A/T1007A mutations on social behaviors, we also detected deficient or improved memory retention in the S940A and T906A/T1007A mice, respectively. The contrasting impact of the S940A and T906A/T1007A mutations on memory retention detected in this study is interesting in light of the fact that both deficient and improved memory retention is associated with autism-spectrum disorders (Gras-Vincendon et al., 2008; Treffert, 2009). Our observation that S940A mice show deficits in spatial memory retention suggests that there may be a pathological link between the delayed E_GABA_ shift detected in several neurodevelopmental disorders and the often co-morbid intellectual disability present in these patients. Interestingly, several studies suggest that adulthood regulation of KCC2 can impact memory retention. Reduced KCC2 expression and depolarizing GABA has been detected in aged mouse brains, which is suggested to reduce synapse specificity of LTP and contribute to cognitive decline in old age (Ferando et al., 2016). Moreover, KCC2 deficits have been detected in several neurodegenerative disorders (Fuchs et al., 2010) and reducing intracellular Cl^−^ levels can rescue memory deficits in a mouse model of Huntington’s disease (Dargaei et al., 2018), suggesting that increasing KCC2 function may be therapeutically beneficial for disorders associated with memory deficits. In support of this, T906A/T1007A mice performed better in the spatial memory tasks compared to their WT littermates, suggesting that increasing KCC2 function may improve memory retention.

It is possible that rapid transient neuronal Cl^−^ loading may play a role in memory retention, rather than a specific impact of altered GABAergic signaling during development. Adulthood manipulation of KCC2 function would be required to differentiate the developmental vs. the mature alteration in KCC2 function as the cause of the observed differences in memory retention between the S940A and T906A/T1007A mutant mice. Moreover, whether autism-associated memory impairments can be rescued in adulthood through KCC2 manipulation, or whether any potential benefit of enhancing KCC2 function is limited to a critical period during development, is unknown; a lack of pharmacological KCC2 activators prevents this question from being addressed (Cardarelli et al., 2017).

We do not know how these developmental changes in KCC2 function could lead to altered cognitive function, but we can speculate that aberrant network formation during a critical period of brain development is responsible. Interestingly, premature E_GABA_ hyperpolarization through KCC2 overexpression in cortical neurons impairs their morphological maturation (Cancedda et al., 2007). Additionally, premature overexpression of KCC2 results in a permanent decrease in excitatory synaptic signaling (Akerman and Cline, 2006). However, many previous studies have established a role for KCC2 in regulating spine formation and maturation through a mechanism independent of its transport function and is instead regulated by KCC2 protein expression levels (Li et al., 2007; Gauvain et al., 2011; Fiumelli et al., 2013; Blaesse and Schmidt, 2015; Llano et al., 2015; Awad et al., 2018). As S940A and T906A/T1007A neurons have comparable KCC2 protein expression to WT neurons at early and late developmental periods (Silayeva et al., 2015; Moore et al., 2018) it is possible that neuronal morphology and synapse formation would not be impacted in this same way and therefore is likely not responsible for the behavioral changes seen in these KCC2 phospho-mutant mice. However, we have not examined whether the S940A or T906A/T1007A mutations impact KCC2 surface stability at the early postnatal period as we were not able to generate enough material to measure this parameter at early time points using biotinylation. Still, it is important to note that published studies have shown that mutation of either S940 or T906/1007 do not alter plasma membrane levels of KCC2 in mature neurons (Silayeva et al., 2015; Moore et al., 2018), which suggests that the impact of the respective mutations on Cl^−^ levels is not related to KCC2 surface expression levels *per se*. This could also explain why we do not see any changes in motor learning in our phospho-mutant mice (Silayeva et al., 2015; Moore et al., 2018) despite a previous study documenting that rates of motor learning are improved when KCC2 is overexpressed (Nakamura et al., 2019).

Blocking early depolarizing GABA using a pharmacological inhibitor of NKCC1 also impairs excitatory synaptic signaling (Akerman and Cline, 2006; Wang and Kriegstein, 2011). However, as NKCC1 is also present in glia, deficits in glial function may be responsible for these synaptic abnormalities rather than a specific impact of altered Cl^−^ homeostasis. Interestingly, however, migrating interneurons expressing KCC2 show reduced motility in response to GABA application (Bortone and Polleux, 2009) suggesting that higher levels of KCC2 function can terminate interneuron migration which supports a role for the Cl^−^ transport function of KCC2 in neuronal network formation. It is, therefore, possible that premature/delayed termination of interneuron migration may occur in the T906A/T1007A and S940A mice, respectively; as autism-spectrum disorders have been associated with altered interneuron placement this would be an interesting question to address (Katsarou et al., 2017). It is also important to consider that these KCC2 mutations may be altering GABAergic signaling in newborn granule cells in the dentate gyrus of the adult hippocampus, as knocking-down NKCC1 results in a premature hyperpolarizing onset of hyperpolarizing GABA currents in the newborn neurons in the adult brain which leads to defective dendritic development and synapse formation (Ge et al., 2006). However, altered NKCC1 function in glia may be responsible for these dendritic and synaptic deficits.

Ultimately, this work suggests that KCC2 phosphorylation and thus the polarity of synaptic inhibition is finely orchestrated during a critical period of postnatal development, which may be necessary for ensuring normal brain function in adulthood. These findings further our insight into molecular events that regulate KCC2 function and GABA_A_ receptor activity that may go awry in autism-spectrum disorders, and suggest a potential role of altered timing of postnatal E_GABA_ hyperpolarization in the behavioral manifestations of these disorders. Examining the phosphorylation state of KCC2 in neurodevelopmental disorders would, therefore, be informative. How social behaviors and memory retention are specifically vulnerable to altered KCC2 function in the phospho-mutant mice, despite other behaviors such as motor function and anxiety-like behaviors being unaffected (Silayeva et al., 2015; Moore et al., 2018), also requires further investigation but may be due to differential regulation of KCC2 phosphorylation in different brain regions. We chose to perform our biochemical and electrophysiological studies on hippocampal neurons as these are some of the most homogeneous neuronal populations in the brain and would, therefore, avoid any variability that may result from exploring other brain regions which are composed of a much more heterogeneous population of neurons. Additionally, given the known role of the hippocampus for spatial memory, the hippocampus is potentially the most relevant region of the brain to examine for our study. Examination of additional brain regions would indeed be interesting in light of our identification of social interaction abnormalities, but the array of different brain regions contributing to social behavior, including PFC, Amygdala, VTA and Accumbens (Gunaydin et al., 2014), make this a large undertaking.

We propose that potentiating KCC2 function during development to rescue the delayed E_GABA_ shift detected in several neurodevelopmental disorders (Duarte et al., 2013; He et al., 2014; Deidda et al., 2015; Tang et al., 2016) may alleviate the complex cognitive deficits characteristic of these disorders. This adds to the already promising prospects of KCC2 activators for other neurological disorders such as for the treatment of seizures (Moore et al., 2017, 2018), a common co-morbidity of autism-spectrum disorders. Further investigations into the signaling cascades that regulate KCC2 phosphorylation may help identify novel therapeutic targets for these disorders. However, given our finding that loss of depolarizing GABA in development disrupts establishment of normal social behavior, caution may be needed in increasing KCC2 function in healthy children to prevent complete elimination of depolarizing GABA in the immature brain. Nevertheless, this work suggests that KCC2 may be a promising novel therapeutic target for alleviating some of the symptoms of these complex disorders.

## Ethics Statement

All mice were handled according to protocols approved by the Institutional Animal Care and Use Committee (IACUC).

## Author Contributions

YM, NB, TD and SM designed the research. YM, LC and HW performed the research. YM, LC and TD analyzed the data. YM, TD and SM wrote the article.

## Conflict of Interest Statement

NB and HW were full-time employees and shareholders of AstraZeneca at the time the studies were conducted. SM serves as a consultant for SAGE therapeutics and AstraZeneca, relationships that are regulated by Tufts University. The remaining authors declare that the research was conducted in the absence of any commercial or financial relationships that could be construed as a potential conflict of interest.
