# Supplementary material for: Developmental Regulation of KCC2 Phosphorylation Has Long-Term Impacts on Cognitive Function
Source: Front Mol Neurosci. 2019 Jul 23;12:173. doi: 10.3389/fnmol.2019.00173 (PMC6664008; doi:10.3389/fnmol.2019.00173)
Supplement: Supplementary file 1 [file Table_1.docx]

**Supplementary material**

**Figure S1. Neuronal resting membrane potentials (RMP) are unaffected by S940A and T906A/T1007A mutations at all developmental stages.** RMP measurements from WT, S940A and T906A/T1007A neurons between 4 and 22 DIV, along with data binned into groups of 2-4 DIV to enable statistical analysis.

**Figure S2. Total KCC2 protein expression levels are not impacted by S940A or T906A/T1007A mutations in the early postnatal period.** Total KCC2 expression in WT, T906A/T1007A and S940A hippocampi at postnatal day 5 was assessed using western blotting. KCC2 levels were normalized to the loading control, β-actin. No significant differences in KCC2 expression were detected between the genotypes.

**Supplementary table 1.** E_GABA_ values obtained from WT, S940A and T906A/T1007A hippocampal neurons between 4 – 22 DIV, binned into groups of 2-4 DIV. The P values for each developmental time point are shown. * represents significance.

| **Days *in vitro*** | **WT E_GABA_ mean ± SEM (mV)** | **WT N** | **S940A E_GABA_ ± SEM (mV)** | **S940A N** | **T906A/T1007A E_GABA_ ± SEM (mV)** | **T906A/T1007A N** | **P value (WT vs S940A)** | **P value (WT vs T906A/T1007A)** |
| --- | --- | --- | --- | --- | --- | --- | --- | --- |
| **4-6** | -46 ± 2 | 15 | -32 ± 1 | 7 | -62 ± 3 | 16 | *** < 0.0001** | *** < 0.0001** |
| **7-9** | -48 ± 2 | 13 | -46 ± 5 | 8 | -88 ± 3 | 21 | 0.6131 | *** < 0.0001** |
| **10-13** | -58 ± 2 | 27 | -49 ± 4 | 9 | -101 ± 3 | 14 | 0.0612 | *** < 0.0001** |
| **14-16** | -70 ± 2 | 19 | -56 ± 2 | 13 | -109 ± 4 | 5 | *** < 0.0001** | *** < 0.0001** |
| **18-19** | -89 ± 3 | 11 | -55 ± 2 | 9 | -111 ± 5 | 3 | *** < 0.0001** | *** 0.0043** |
| **21-22** | -81 ± 4 | 11 | -74 ± 4 | 4 | -112 ± 2 | 8 | 0.3890 | *** < 0.0001** |

**Supplementary table 2.** [Cl^–^]_i_ values obtained from WT, S940A and T906A/T1007A hippocampal neurons between 4 – 22 DIV, binned into groups of 2-4 DIV. The P values for each developmental time point are shown. * represents significance.

| **Days *in vitro*** | **WT [Cl**^–^**]i mean ± SEM (mM)** | **WT N** | **S940A [Cl**^–^**]i mean ± SEM (mM)** | **S940A N** | **T906A/T1007A [Cl**^–^**]i mean ± SEM (mM)** | **T906A/T1007A N** | **P value (WT vs S940A)** | **P value (WT vs T906A/T1007A)** |
| --- | --- | --- | --- | --- | --- | --- | --- | --- |
| **4-6** | 26 ± 2 | 15 | 43 ± 2 | 7 | 15 ± 2 | 16 | *** < 0.0001** | *** 0.0004** |
| **7-9** | 24 ± 2 | 13 | 28 ± 4 | 8 | 6 ± 1 | 21 | 0.2655 | *** < 0.0001** |
| **10-13** | 17 ± 1 | 27 | 24 ± 3 | 9 | 3 ± 0 | 14 | *** 0.0335** | *** < 0.0001** |
| **14-16** | 10 ± 1 | 19 | 17 ± 2 | 13 | 2 ± 0 | 5 | *** < 0.0001** | *** < 0.0001** |
| **18-19** | 5 ± 0 | 11 | 17 ± 1 | 9 | 2 ± 0 | 3 | *** < 0.0001** | *** 0.0143** |
| **21-22** | 7 ± 1 | 11 | 8 ± 2 | 4 | 2 ± 0 | 8 | 0.4618 | *** 0.0004** |

**Supplementary table 3.** Resting membrane potential (RMP) values obtained from WT, S940A and T906A/T1007A hippocampal neurons between 4 – 22 DIV, binned into groups of 2-4 DIV. The P values for each developmental time point are shown.

| **Days *in vitro*** | **WT RMP mean ± SEM (mV)** | **WT N** | **S940A RMP mean ± SEM (mV)** | **S940A N** | **T906A/T1007A RMP mean ± SEM (mV)** | **T906A/T1007A N** | **P value (WT vs S940A)** | **P value (WT vs T906A/T1007A)** |
| --- | --- | --- | --- | --- | --- | --- | --- | --- |
| **4-6** | -43 ± 2 | 15 | -40 ± 2 | 7 | -44 ± 2 | 16 | 0.4024 | 0.7265 |
| **7-9** | -48 ± 3 | 13 | -48 ± 3 | 8 | -45 ± 2 | 21 | 0.8976 | 0.3452 |
| **10-13** | -52 ± 2 | 27 | -51 ± 2 | 9 | -51 ± 3 | 14 | 0.8282 | 0.8183 |
| **14-16** | -56 ± 2 | 19 | -59 ± 3 | 13 | -63 ± 4 | 5 | 0.4646 | 0.1803 |
| **18-19** | -58 ± 3 | 11 | -60 ± 3 | 9 | -66 ± 4 | 3 | 0.7052 | 0.3000 |
| **21-22** | -61 ± 2 | 11 | -60 ± 3 | 4 | -61 ± 2 | 8 | 0.6393 | 0.9085 |

**Supplementary table 4.** Latency to goal hole between days 1 and 6 of the barnes maze assay for the S940A mice and their WT littermates. * represents significance.

| **Day** | **WT Latency Mean ± SEM (s)** | **P value (compared to day 1 time)** | **S940A Latency Mean ± SEM (s)** | **P value (compared to day 1 time)** | **P value (between WT and S940A)** |
| --- | --- | --- | --- | --- | --- |
| **1** | 113 ± 21 | NA | 112 ± 17 | NA | \| 0.9548 \| \| --- \| |
| **2** | 100 ± 16 | \| 0.9343 \| \| --- \| | 99 ± 15 | \| 0.7905 \| \| --- \| | \| 0.9645 \| \| --- \| |
| **3** | 84 ± 14 | 0.4465 | 86 ±14 | 0.0623 | \| 0.9199 \| \| --- \| |
| **4** | 80 ± 16 | 0.3519 | 55 ± 12 | *** 0.0126** | \| 0.2164 \| \| --- \| |
| **5** | 65 ± 17 | 0.1745 | 68 ± 15 | 0.1311 | \| 0.8632 \| \| --- \| |
| **6** | 46 ± 9 | *** 0.0384** | 35 ± 10 | *** 0.0008** | \| 0.3928 \| \| --- \| |

**Supplementary table 5.** Latency to goal hole between days 1 and 6 of the barnes maze assay for the T906A/T1007A mice and their WT littermates. * represents significance.

| **Day** | **WT latency Mean ± SEM (s)** | **P value (compared to day 1 time)** | **T906A/T1007A latency Mean ± SEM (s)** | **P value (compared to day 1 time)** | **P value (between WT and T906A/T1007A)** |
| --- | --- | --- | --- | --- | --- |
| **1** | 150 ± 9 | \| NA \| \| --- \| | 159 ± 7 | \| NA \| \| --- \| | \| 0.4127 \| \| --- \| |
| **2** | 126 ± 12 | 0.0806 | 115 ± 12 | *** 0.0094** | 0.5070 |
| **3** | 128 ± 13 | 0.2395 | 112 ± 12 | *** 0.0031** | 0.3706 |
| **4** | 115 ± 11 | *** 0.0427** | 85 ± 13 | *** 0.0002** | 0.0887 |
| **5** | 104 ± 17 | *** 0.0263** | 80 ± 13 | *** 0.0003** | 0.2671 |
| **6** | 60 ± 17 | *** 0.0006** | 57 ± 11 | *** < 0.0001** | 0.8902 |

| **Degrees from goal** | **WT time Mean**  **± SEM (s)** | **WT P value (compared to 0˚)** | **S940A time Mean ± SEM (s)** | **S940A P value (compared to 0˚)** | **P value (WT vs S940A)** | **WT 2 Mean ± SEM (s)** | **WT 2 P value (compared to 0˚)** | **T906A/**  **T1007A time Mean ± SEM (s)** | **T906A/T1007A P value (compared to 0˚)** | **P value (WT 2 vs T906A/**  **T1007A)** |
| --- | --- | --- | --- | --- | --- | --- | --- | --- | --- | --- |
|  | **Day 7** | | | | | | | | | |
| **180** | 6 ± 1 | *** 0.0011** | 7 ± 1 | 0.0869 | 0.6770 | 8 ± 3 | 0.0954 | 5 ±1 | *** < 0.0001** | 0.3242 |
| **225** | 5 ± 1 | *** 0.0015** | 6 ± 1 | *** 0.0260** | 0.8216 | 6 ± 1 | *** 0.0003** | 6 ±1 | *** < 0.0001** | 0.9851 |
| **270** | 6 ± 1 | *** 0.0036** | 6 ± 1 | *** 0.0115** | 0.6981 | 6 ± 1 | *** 0.0016** | 7 ±1 | *** = 0.0001** | 0.5954 |
| **315** | 6 ± 1 | *** 0.0015** | 5 ± 1 | *** 0.0067** | 0.5928 | 4 ± 1 | *** < 0.0001** | 4 ±1 | *** < 0.0001** | 0.7008 |
| **0** | 17 ± 2 | NA | 14 ± 3 | NA | 0.3526 | 15 ± 1 | NA | 20 ±2 | NA | *** 0.0279** |
| **45** | 6 ± 1 | *** 0.0015** | 5 ± 1 | *** 0.0009** | 0.3380 | 4 ± 1 | *** < 0.0001** | 4 ±1 | *** < 0.0001** | 0.9047 |
| **90** | 6 ± 1 | *** 020009** | 7 ± 1 | 0.0835 | 0.6515 | 8 ± 2 | *** 0.0156** | 7 ±1 | *** < 0.0001** | 0.4728 |
| **135** | 5 ± 1 | *** 0.0003** | 5 ± 1 | *** 0.0322** | 0.8005 | 3 ± 1 | *** < 0.0001** | 3 ±1 | *** < 0.0001** | 0.8455 |
|  | **Day 14** | | | | | | | | | |
| **180** | 4 ± 1 | *** 0.0127** | 5 ± 2 | 0.7117 | 0.6724 | 5 ± 1 | *** 0.0039** | 6 ± 1 | *** < 0.0001** | 0.3669 |
| **225** | 3 ± 1 | *** 0.0006** | 5 ± 1 | 0.5215 | 0.2516 | 4 ± 1 | *** 0.0025** | 4 ± 0 | *** < 0.0001** | 0.9222 |
| **270** | 5 ± 1 | *** 0.0078** | 5 ± 1 | 0.7329 | 0.5741 | 7 ± 1 | *** 0.0212** | 7 ± 1 | *** < 0.0001** | 0.9012 |
| **315** | 6 ± 2 | 0.2071 | 5 ± 1 | 0.2831 | 0.4312 | 2 ± 1 | *** 0.0002** | 4 ± 1 | *** < 0.0001** | 0.1164 |
| **0** | 9 ± 2 | NA | 6 ± 1 | NA | 0.1225 | 1 ± 2 | NA | 16 ± 1 | NA | *** 0.0307** |
| **45** | 4 ± 1 | *** 0.0025** | 4 ± 1 | 0.0924 | 0.8286 | 6 ± 2 | 0.0742 | 5 ± 1 | *** < 0.0001** | 0.6973 |
| **90** | 7 ± 2 | *** 0.0390** | 5 ± 1 | 0.5145 | 0.3045 | 6 ± 1 | *** 0.0100** | 5 ± 1 | *** < 0.0001** | 0.5525 |
| **135** | 4 ± 1 | *** 0.0056** | 4 ± 1 | 0.3947 | 0.8869 | 4 ± 1 | *** 0.0015** | 5 ± 1 | *** < 0.0001** | 0.5955 |

**Supplementary table 6.** Time spent at goal area (0˚) and the remaining holes (binned into groups of 45˚) on day 7 and day 14 of the barnes maze for S940A and T906A/T1007A mice and their corresponding WT littermates. * represents significance.
