# Supplementary figures and images for: Developmental Regulation of KCC2 Phosphorylation Has Long-Term Impacts on Cognitive Function
Source: Front Mol Neurosci. 2019 Jul 23;12:173. doi: 10.3389/fnmol.2019.00173 (PMC6664008; doi:10.3389/fnmol.2019.00173)

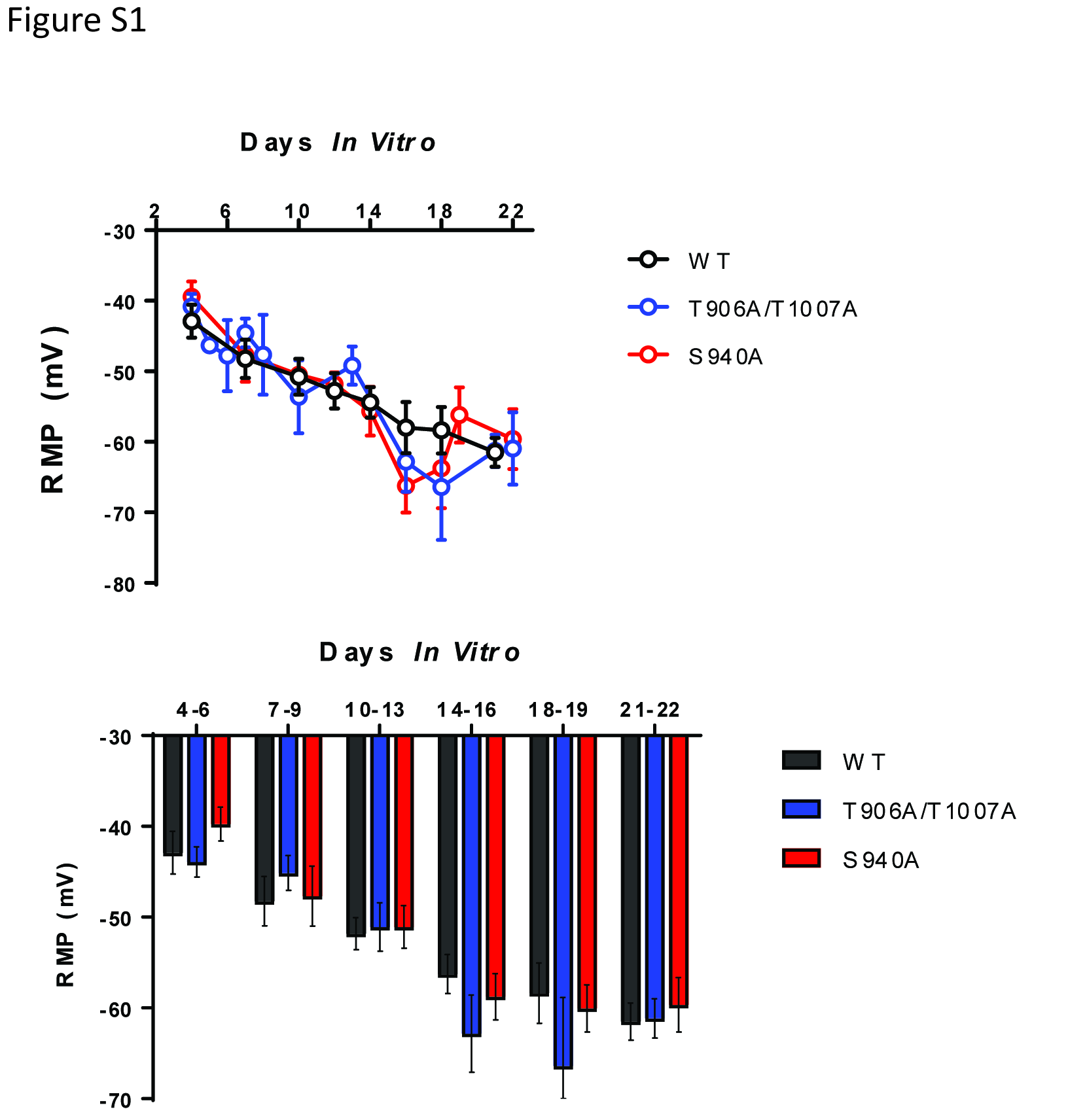

Supplement: Supplementary file 2 [file Image_1.TIF]

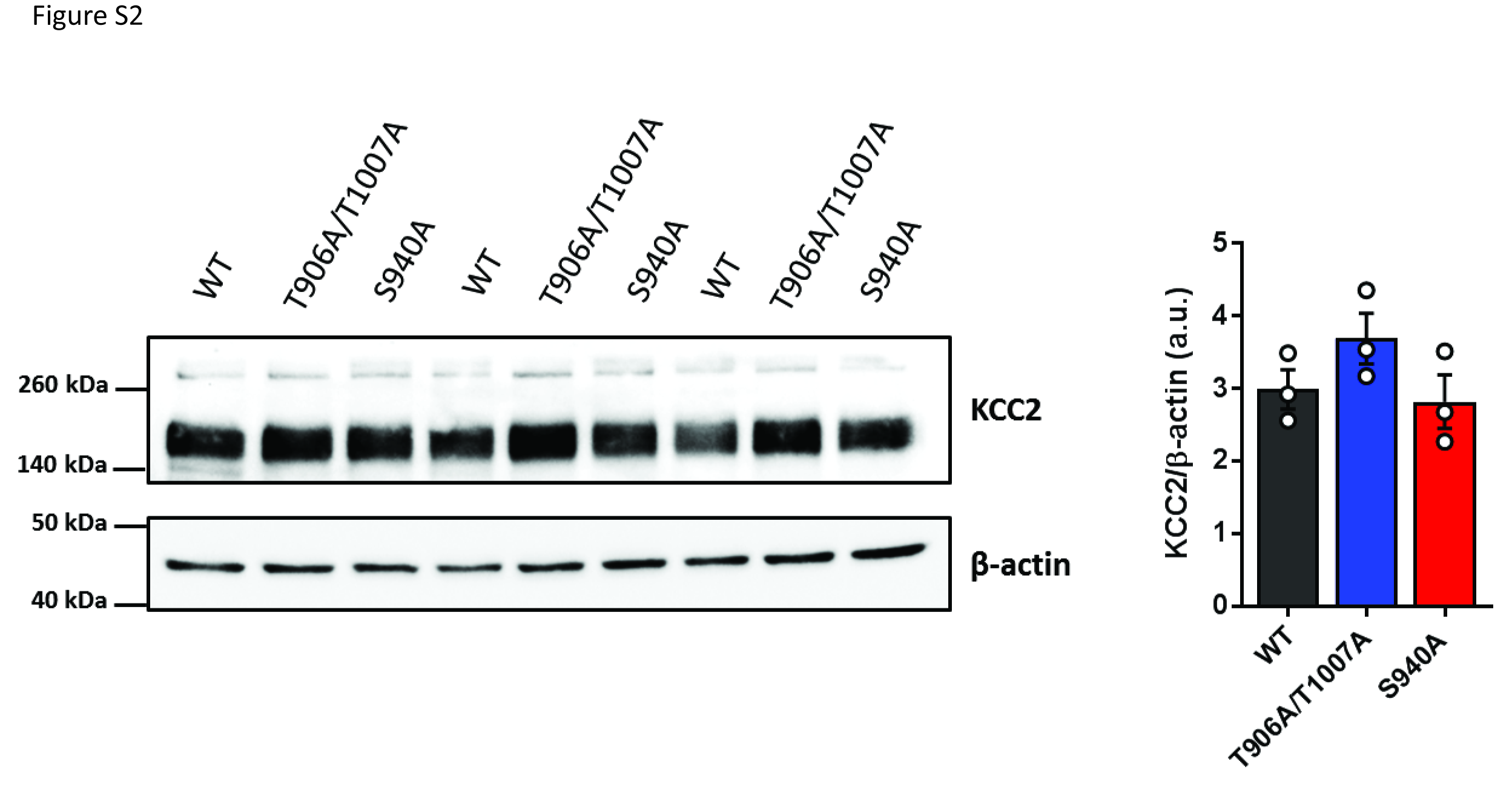

Supplement: Supplementary file 3 [file Image_2.TIF]
